# Molecular characteristics and clinical features of pediatric methicillin-susceptible *Staphylococcus aureus* infection in a medical center in northern Taiwan

**DOI:** 10.1186/s12879-019-4033-0

**Published:** 2019-05-10

**Authors:** Yu-Jen Chen, Po-An Chen, Chih-Jung Chen, Yhu-Chering Huang

**Affiliations:** 1 School of Medicine, Chang Gung University, Kweishan, Taoyuan, Taiwan; 2 Department of Pediatrics, Chang Gung Memorial Hospital at Linkou, No. 5, Fu-Shin Street, Kweishan, 333 Taoyuan, Taiwan

**Keywords:** Methicillin-susceptible *Staphylococcus aureus*, Children, Taiwan

## Abstract

**Background:**

There have been no reports regarding clinical features and molecular characteristics of childhood methicillin-susceptible *Staphylococcus aureus* (MSSA) infections in Taiwan.

**Methods:**

We prospectively collected clinical *S. aureus* isolates from patients aged < 18 years in a university-affiliated hospital in 2015. Only the first isolate from each patient was included. Medical records were retrospectively reviewed. Patients were classified as community-associated (CA) or healthcare-associated (HA) by the epidemiologic criteria. All MSSA isolates were molecularly characterized.

**Results:**

A total of 495 *S. aureus* isolates were identified, and 178 (36.0%) were MSSA. Among the 131 MSSA-infected patients enrolled, 94 (71.8%) were community-associated and 60 (45.8%) were inpatients. Patients with HA infections was significantly younger than those with CA infections (median, 15 vs. 67.5 months). The most common specimen of MSSA identified was pus or wound (73.3%). Compared to HA-MSSA, CA-MSSA isolates were significantly less frequently from sputum (6.4% vs. 27%, *p* = 0.001). Nineteen pulsotypes were identified. Four pulsotypes accounted for 60% of the isolates. Isolates of ST15/pulsotype F were more frequently seen in CA than in HA (*p* = 0.064) while isolates of ST188/pulsotype AX frequently seen in HA (*p* = 0.049). PVL genes were identified in 11 isolates (8.4%), nine of which were characterized as ST59/pulsotype D, same as the local endemic CA-MRSA clone.

**Conclusions:**

MSSA accounted for around one-third of childhood *S. aureus* infections in northern Taiwan. SSTI was the most common manifestation. The molecular characteristics of these clinical MSSA isolates were relatively diverse and had certain significant differences between CA and HA isolates.

## Background

*Staphylococcus aureus* is among the most commonly isolated human pathogens both in the community and in the hospital setting [1].The pathogen can be frequently found in the nose, throat, and on the skin as normal flora [2]. Although *S. aureus* is not always pathogenic, it can cause a variety of infection**s** in human, including skin-soft tissue infection, respiratory tract infection, pneumonia, bone/joint infection, endocarditis, sepsis, and toxic shock syndrome [3].

MRSA (methicillin-resistant *S. aureus*) has traditionally been a hospital acquired pathogen and became an increasingly common cause of community-onset infections in the 1990s [4]. However, infections caused by methicillin-susceptible *S. aureus* (MSSA) are still much more frequent than MRSA [5]. Recent studies report that the incidence of MRSA has decreased since the late 2000s [6], while MSSA infections have increased or at least remained stable [7, 8]. In Taiwan, incidence of MSSA infection was reported to be 0.132 episode per 100 discharges in 2007 [9] and our previous report showed that MSSA accounted for one-third of childhood *S. aureus* infection between 2006 and 2012 in a medical center situated in northern part of Taiwan [10]. Some studies from Europe found that the overall annual incidence rate of MSSA bacteremia was 10-fold higher than that of MRSA bacteremia from 2000 to 2008 [8]. Therefore, the epidemiology of MSSA is as important as MRSA nowadays.

In Taiwan, MRSA has prevailed more common than MSSA clinically and has attracted more attention and studies [9, 10]. Studies regarding the clinical features as well as molecular characteristics of MSSA infections have been scanty [11, 12]**,** all focusing on adult patients, and none from pediatric patients. Therefore, we conducted this study to figure out the clinical features and molecular characteristics of pediatric MSSA infections in Taiwan.

## Methods

### Population

This study was conducted in Chang Gung Memorial Hospital (CGMH) at Linkou, which is a university-affiliated teaching hospital in northern Taiwan and provides a range of care, from primary to tertiary care, with 3700 beds. From January 1, 2015 to December 31, 2015, all the clinical *S. aureus* isolates from children less than 18 years of age, excluding the isolates from neonatal units and survey for colonization, were prospectively collected and stored by the microbiology laboratory of CGMH. For MSSA isolates, we retrospectively reviewed the medical records of the patients. If there were multiple episodes (isolates) collected from a single patient, only the first episode (isolate) was included for analysis. We classified MSSA-infected patients as healthcare-associated (HA) and community-associated (CA) using the Centers for Disease Control and Prevention definition for MRSA [3, 13, 14]. “HA” infection was defined as the patient with MSSA infection identified 48 h after hospitalization or having prior healthcare exposure history within 1 year prior to the episode, including residence in a long-term care facility, prior admission to an acute care facility, prior surgical procedures, the use of central intravenous catheters or long-term venous access devices, the use of urinary catheters, the use of other long-term percutaneous devices, and/or the requirement of dialysis. “CA” infection was defined as the patient having MSSA infection identified within 48 h of hospitalization and without aforementioned history within 1 year before the episode. HA infection was further categorized into hospital-onset (HO) and community-onset (CO) based on the timing of specimens collected. The samples collected at outpatient department or within 48 h of hospitalization were categorized as CO [15] and those collected after 48 h of hospitalization were categorized as HO [10, 16].

The study was approved by the Institutional Review Board (IRB) of CGMH and informed consents from the patients/guardians were waived.

### Definition of the diagnosis

Primary bacteremia was defined as blood cultures positive for MSSA without apparent infectious foci. The diagnosis of pneumonia composed of clinical symptoms (fever with respiratory symptoms) and a radiographic confirmation [17]. Urinary tract infection (UTI) was defined as a significant bacteriuria (≥100,000 colony forming units from a cleanly voided sample or > 50,000 colony forming units from catheter sample [18]) with pyuria [19]. Those with colonies of MSSA isolates not reaching the amount as significant bacteriuria (≤ 100,000 colony forming units from a cleanly voided sample or < 50,000 colony forming units from catheter sample) were excluded for further analysis.

### Antibiotic susceptibility testing

The antimicrobial susceptibility of all MSSA isolates to 10 antibiotics, including penicillin, trimethoprim/sulfamethoxazole (SXT), ciprofloxacin, clindamycin, erythromycin, doxycycline, fusidic acid, teicoplanin and linezolid, was determined by the disk-diffusion method, while vancomycin was determined by minimal inhibition concentration, following the 2016 Guideline of Clinical and Laboratory Standard Institutes [20].

### Molecular characterizations

Molecular methods used included pulsed-field gel electrophoresis (PFGE) by *Sma*I digestion and detection of the Panton-Valentine leukocidin (PVL) genes as previously described [10]. Some isolates of representative PFGE patterns were selected for further characterization by multilocus sequence typing (MLST), and *spa* typing. Sequence type (ST) was given according to sequence allelic profiles using the MLST database (https://pubmlst.org/saureus/) [21]. The *spa* genes were sequenced and *spa* types were assigned corresponding to the *spa* database (http://spa.ridom.de/spatypes.shtml).

### Statistics

The associations between the clinical presentations of MSSA-infected patients among the two groups were compared using the Kruskal–Wallis one-way analysis of variance (ANOVA) or Fisher’s exact test. A *p* value of less than 0.05 was considered significant. All analyses were performed by SPSS 17.0 for Windows.

## Results

### Patient characteristics and infection foci

During the study period, a total of 495 *S. aureus* isolates were included, and 178 (36.0%) from 160 patients were MSSA.14 patients had multiple isolates, including 11 patients with 2 isolates, two patients with three isolates and one patient with four isolates. Among the 160 patients enrolled, 29 patients with bacteriuria were excluded and a total of 131 patients were finally included for analysis. Of the 131 patients, 94 (71.8%) were classified as CA and 37 (28.2%) as HA. The distribution of these patients is shown in Table 1. Seventy-one patients (54.2%) were treated as outpatients or visitors of emergency department (OPD/ED), whereas 60 (45.8%) were from inpatients. Among the 71 OPD/ED, 65 (91.5%) were CA and only 6 as HA. Patients with HA infection were younger than those with CA (median age, 15 months (4–156) vs. 67.5 months (22.3–165)). The patients with HA infection were significantly more likely to have underlying diseases, and among the listed underlying diseases, more likely to be preterm patients and have neurological problems such as cerebral palsy and epilepsy (all *P* < 0.05).

**Table 1:** Comparison of demographics and clinical outcomes of pediatric patients with MSSA Infection stratified by the origin of infections

Characteristics	CA (*n* = 94) No. (%)	HA (*n* = 37) No. (%)	*P* value	HA	
				CO (*n* = 17) No. (%)	HO (*n* = 20) No. (%)
Demographics					
Age in month median, IQR)	67.5 (22.3–165)	15 (4–156)		66 (5.5–168.5)	6 (3–75)
Male sex (male%)	63 (67)	21 (56.8)	0.270	10 (58.8)	11 (55)
Underlying Diseases					
None (%)	61 (46.6)	15 (40.5)	0.011	6 (35.3)	9 (45)
Atopic syndrome (%)	14 (10.7)	1 (2.7)	0.049	0 (0)	1 (5)
Preterm (%)	0 (0)	7 (18.9)	0	0 (0)	7 (35)
Immunodeficiency (%)	0 (0)	1 (2.7)	0.110	1 (5.9)	0 (0)
Renal Diseases (%)	1 (1)	1 (2.7)	0.491	1 (5.9)	0 (0)
Urogenital problems (%)	0 (0)	2 (5.4)	0.023	1 (5.9)	1 (5)
Gastrointestinal problems (%)	0 (0)	1 (2.7)	0.110	0 (0)	1 (5)
Cardiovascular diseases (%)	1 (1)	1 (2.7)	0.491	1 (5.9)	0 (0)
Malignancies and Hematologic Diseases (%)	0 (0)	2 (5.4)	0.023	1 (5.9)	1 (5)
Metabolic Diseases (%)	2 (1.5)	1 (2.7)	0.843	0 (0)	1 (5)
Neurological Diseases (%)	5 (3.8)	7 (18.9)	0.015	7 (41.2)	0 (0)
ENT anomaly (%)	12 (9.2)	3 (8.1)	0.451	3 (17.6)	0 (0)
Inpatient (%)	29 (22.1)	31 (83.8)	0	11 (64.7)	20 (100)
Outcome					
Septic shock (%)	1 (0.8)	0 (0)			
Attributable mortality (%)	0 (0)	0 (0)			
Non-attributable mortality (%)	0 (0)	3 (8.1)			
Recurrent infection (%)	3 (2.3)	0 (0)			
Uneventfully (%)	88 (93.6)	34 (91.9)			

*CA* community-associated, *HA* healthcare-associated, *CO* community onset, *HO* hospital onset

The detailed distribution of the specimens of these MSSA isolates identified is shown in Table 2. Pus or wound accounted for the largest proportion of both CA and HA patients (79.8 and 56.8%, respectively). Compared to HA-MSSA, CA-MSSA isolates were significantly less frequently identified from sputum (6.4% vs. 27.0%, *p* = 0.001) while significantly more frequently identified from pus or wound (*P* = 0.007).

**Table 2:** Distribution of the specimens of 131 MSSA isolates identified from pediatric patients

Types of specimens	Inpatients (*n* = 60) No. (%)	Outpatients (*n* = 71) No. (%)	CA (n = 94) No. (%)	HA (n = 37) No. (%)	P	HA	
						CO (*n* = 17) No. (%)	HO (*n* = 20) No. (%)
Bloodstream (%)	2 (3.3)	6 (8.5)	6 (6.4)	2 (5.4)	0.833	0 (0)	2 (10)
Central venous catheter (%)	2 (3.3)	0 (0)	0 (0)	2 (5.4)	0.023	2 (11.8)	0 (0)
Sputum (%)	15 (25)	1 (1.4)	6 (6.4)	10 (27)	0.001	5 (29.4)	5 (25)
Pus or wound (%)	35 (58.3)	61 (85.9)	75 (79.8)	21 (56.8)	0.007	9 (52.9)	12 (60)
Urine (%)	1 (1.7)	0 (0)	0 (0)	1 (2.7)	0.110	0 (0)	1 (5)
Ear discharge (%)	4 (6.7)	3 (4.2)	6 (6.4)	1 (2.7)	0.399	1 (5.9)	0 (0)
Synovial fluid (%)	1 (1.7)	0 (0)	1 (1)	0 (0)	0.529	0 (0)	0 (0)

*CA* community-associated, *HA* healthcare-associated, *CO* community onset, *HO* hospital onset

### Clinical presentations of MSSA infections

Clinical manifestations of these MSSA infections included SSTIs (*n* = 95), pneumonia (*n* = 16), primary bacteremia (*n* = 8), otitis media (*n* = 7), central catheter infection (*n* = 2), osteomyelitis (*n* = 1), septic arthritis (*n* = 1), and UTI (*n* = 1). More than 90% of the cases recovered uneventfully. One developed septic shock. Four patients eventually died during hospitalization but were not attributed to MSSA infections.

Skin and soft tissue infections were the most common presentation for both CA and HA MSSA infections. Thirty-five out of 95 were hospitalized (36.8%). The outcomes were generally uneventful. Two patients had two isolates within 14 days. The first case was an 8-year-old boy without underlying disease who suffered from otitis externa initially. Recurrent infection of otitis externa was noted after one course of antibiotics treatment and MSSA was identified from the pus again. The other case was a 2-year-old girl without underlying disease. She was diagnosed as pre-auricular fistula in the first visit. After antibiotics treatment for 2 weeks, pre-auricular abscess was still noted and the second pus specimen still yielded MSSA.

Pneumonia occurred in 16 children. Fifteen of them were inpatients and five of them were CA (33.3%). The average hospital stay of these patients was 87.3 days (61.6 days for CA cases and 100.4 days for HA cases), which was the longest of all the infections. One of them experienced a complicated clinical course and MSSA non-attributable death occurred in three patients. A 12-month-old male had MSSA pneumonia, received a 10-day course of antibiotic treatment and was discharged. Lung abscess and septic shock was noted 3 days after discharge and re-admitted for a complete course of intravenous antibiotics therapy. Two preterm infants suffering from respiratory distress syndrome and severe bronchopulmonary dysplasia had MSSA isolates identified from sputum during hospitalization. One died later due to pneumonia caused by *Acinetobacter nosocomialis* and the other due to severe bronchopulmonary dysplasia. The third case, a 4-month-old male infant without systemic disease, encountered a life-threatening event, was intubated, MSSA identified from the sputum during hospitalization and eventually died.

Primary bacteremia occurred in 8 children and only two of them was inpatient. Both inpatients were grouped in HA (HO)-MSSA. Central catheter-related infection was identified in two inpatients. All of them recovered uneventfully.

Osteomyelitis occurred in a 17-year-old adolescent, who suffered from an open fracture after a traffic accident, MSSA identified from chronic wound and recovered uneventfully after antibiotic treatment. Septic arthritis occurred in one inpatient with uneventful outcome after a course of antibiotic regimen.

### Molecular typing

**Fig. 1:**
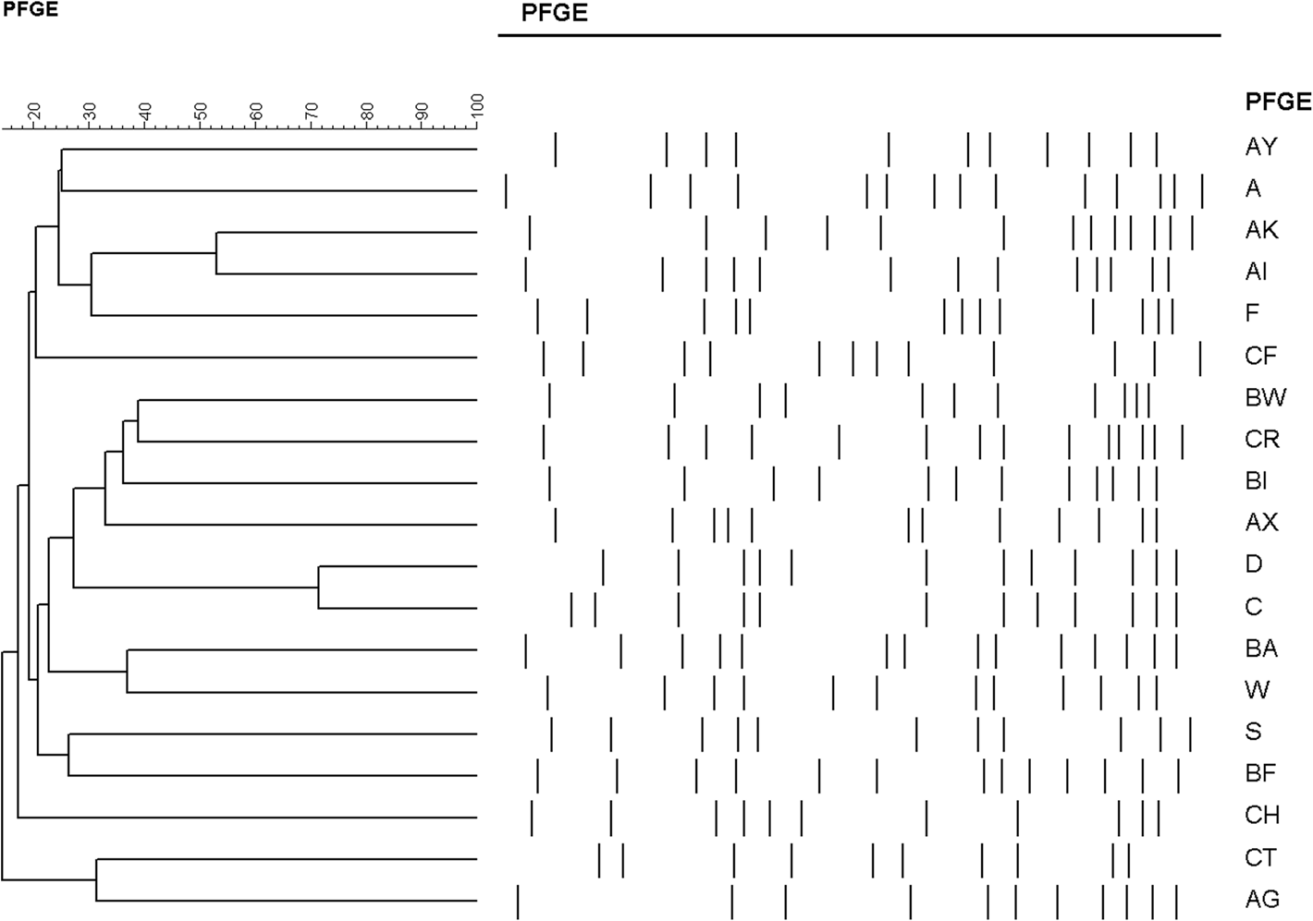
Dendrogram of pulsed-field gel electrophoresis (PFGE) cluster analysis of 131 methicillin-sensitive *Staphylococcus aureus* isolates

Detailed molecular characteristics of the 131 isolates including PFGE, MLST (performed for selected isolates) and *spa* typing (performed for selected isolates) are shown in Table 3. A dendrogram for all 19 pulsotypes with the cut-off value is provided in Fig. 1. Except three untypeable isolates, nineteen pulsotypes were identified. Four pulsotypes, including types BA (18.3%), AX (16.8%), F (14.5%) and BW (10.7%), accounted for 60.3% of the 131 isolates. Pulsotype AX accounted for a borderline significantly higher proportion of HA isolates (27%) than CA isolates (12.8%) (*p* = 0.049) while pulsotype F accounted for a higher proportion of CA isolates (18.1%) than HA isolates (5.4%) but did not reach a significant difference (*p* = 0.064). MLST typing was performed in 44 selected samples among each PFGE type and except for five untypeable isolates a total of 20 sequence types were identified, with singletons for 9 isolates. For pulsotype AX, all five isolates selected showed ST188 (5/5). For pulsotype BA and F, ST7 (4/6) and ST15 (6/6) are dominant MLST types, respectively. As for *spa* typing, 56 isolates were analyzed and except for five untypeable isolates 32 *spa* types were identified with at least three newly identified types. Nine of 22 PFGE type AX isolates were analyzed, and all of them presented *spa* t189. Generally, isolates of ST15/pulsotype F were more frequently seen in CA than in HA with borderline significance (*P* = 0.064) while isolates of ST188/pulsotype AX were significantly frequently seen in HA (*p* = 0.049).

**Table 3:** Molecular characteristics of 131 methicillin-sensitive *Staphylococcus aureus* isolates from pediatric patients stratified by pulsotypes

Characteristics	BA	AX	F	BW	AK	D	S	Others
No. isolates (*n* = 131)	24 (18.3%)	22 (16.8%)	19 (14.5%)	14 (10.7%)	10 (7.6%)	9 (6.9%)	7 (5.3%)	26 (19.8%)
CA (*n* = 94)	18 (19.1%)	12 (12.8%)	17 (18.1%)	10 (10.6%)	9 (9.8%)	5 (5.3%)	6 (6.4%)	17 (18.1%)
HA (*n* = 37)	6 (16.2%)	10 (27.4%)	2 (5.4%)	4 (10.8%)	1 (2.7%)	4 (10.8%)	1 (2.7%)	9 (24.3%)
*p*-value	0.696	0.049	0.064	0.977	0.182	0.263	0.399	0.420
PVL-positive (*n* = 11)	0	0	0	0	0	9	0	2
Sequence type	7(4/6), 8(1/6), 6(1/6)	188(5/5)	15(6/6)	nontypeable (3/3)	508 (3/5), 3563 (1/5), 3375 (1/5)	59 (2/2)	5 (2/2)	1281 (2), nontypeable (2), 1, 8, 12, 30, 59, 182, 398, 573, 623, 834, 1301
*spa* type	t091(2/9), t008(1/9), t701(1/9), t1943(1/9), t3071(1/9), t3864(1/9), t3992(1/9), t16261(1/9)	t189(9/9)	t084 (4/9), t7200(2/9), t346(1/9), t3024(1/9), t16565(1/9)	nontypeable (2/2)	t015 (2/6), t116 (2/6), t073 (1/6), t16564 (1/6)	t437(2/3), t1950(1/3)	t002 (2/3), nontypeable (1/3)	nontypeable (2), t008, t012, t164, t213, t364, t437, t1250, t1379, t2182, t3406, t8940, t16297, t16298

*PVL* Panton-Valentine leucocidin genes

The isolates unable to be successfully typed by MLST were clustered in two pusotypes, namely type BW and BI. All 14 isolates with pulsotype BW were re-examined by biochemistry tests and MALDI-TOF (matrix-assisted laser desorption/ionization time0of-flight mass spectrometer) and were confirmed as *S. aureus*. Three isolates were selected for MLST and 4 of 7 segments (*arc, gmk, pta, tpi*) could be amplified and sequenced successfully, all revealing the same sequences. However, the other three segments could not be either amplified or sequenced successfully and thus was categorized as untypeable.

PVL genes were identified in 11 isolates (8.4%), and nine of them were characterized as ST59/pulsotype D, same as the local endemic CA-MRSA clone in Taiwan. Nine of them were associated with SSTI and seven patients were male (Table 4).

**Table 4:** Clinical and molecular features of the 11 patients with Panton-Valentine leucocidin-positive MSSA isolates

Age range (years)	Gender	IP/OP	Underlying diseases	Diagnosis	Onset Group	Outcome	PFGE	MLST-spa
5–10	F	OP	None	Primary bacteremia	CA	Uneventfully	CT	ST1301-t8940
< 1	M	IP	None	SSTI	CA	Uneventfully	AI	ST8-t008
5–10	M	OP	None	SSTI	CA	Uneventfully	D	..
> 10	F	IP	None	SSTI	CA	Uneventfully	D	ST59-t1950
5–10	M	OP	None	SSTI	CA	Uneventfully	D	..
1–4	M	IP	None	SSTI	CA	Uneventfully	D	..
> 10	M	OP	Atopic	SSTI	CA	Uneventfully	D	ST59-t437
> 10	M	OP	None	SSTI	HACO	Uneventfully	D	..
< 1	M	IP	Preterm, Cardiovascular disease	Pneumonia	HO	Non-attributable mortality	D	..
5–10	F	IP	None	SSTI	HO	Uneventfully	D	..
< 1	F	IP	None	SSTI	HO	Uneventfully	D	..

*IP* inpatient, *OP* outpatient, *PFGE* pulsed-field gel electrophoresis, *MLST* multi-locus sequence typing, *CA* community-associated, *HA* healthcare-associated, *CO* community onset, *HO* hospital onset

### Antibiotics sensitivity test

The results of antibiotic susceptibility test are shown in Table 5. All 131 isolates were sensitivity to Linezolid, teicoplanin, and vancomycin, and more than 95% of the isolates were susceptible to TMP-SMX, Fusidic acid, ciprofloxacin and doxycycline. The overall sensitivity rate to clindamycin and erythromycin were 79.4 and 69.5% respectively. The susceptibility rate of HA isolates (22%) to penicillin was significantly higher than that of CA isolates (2.2%) (*p* < 0.01) while CA isolates had significantly higher susceptible rate to TMP-SMX (100%) than did HA isolates (95%) (*p* = 0.006).

**Table 5:** Distribution of antimicrobial susceptibility test results of 131 methicillin-sensitive *Staphylococcus aureus* isolates from pediatric patients stratified by origin of acquisition

Antibiotics	CA (*n* = 94) No. (%)	HA (*n* = 37) No. (%)	P	HA		
				CO (*n* = 17) No. (%)	HO (*n* = 20) No. (%)	*P*
Clindamycin (%)	75 (80)	29 (78)	0.858	12 (71)	17 (85)	0.289
Erythromycin (%)	66 (70)	25 (68)	0.767	11 (65)	14 (70)	0.732
Fusidic acid (%)	93 (99)	35 (95)	0.135	17 (100)	18 (90)	0.180
Linezolid (%)	94 (100)	36/36^a^ (100)	–	17 (100)	19/19^a^ (100)	–
Penicillin (%)	2 (2)	8/36^a^ (22)	0	4 (24)	4/19^a^ (21)	0.858
Sufamethoxazole-Trimethoprim (%)	94 (100)	34/36^a^ (94)	0.021	16 (94)	18/19^a^ (95)	0.935
Teicoplanin (%)	94 (100)	36/36^a^ (100)	–	17 (100)	19/19^a^ (100)	–
Vancomycin (%)	94 (100)	36/36^a^ (100)	–	17 (100)	19/19^a^ (100)	–
iprofloxacin (%)	93 (99)	37 (100)	0.529	17 (100)	20 (100)	–
Doxycycline (%)	92 (98)	35 (95)	0.326	15 (88)	20 (100)	0.115

*CA* community-associated, *HA* healthcare-associated, *CO* community onset, *HO* hospital onset

^a^Linezolid, Penicillin, Sufamethoxazole-Trimethoprim, Teicoplanin, Vancomycin each lost 1 datum in the HA column

## Discussion

To our knowledge, this is the first study regarding the clinical features and molecular characteristics of pediatric MSSA infections in Taiwan. In this study, the most common clinical manifestation was SSTI, which accounted for nearly three quarters of the MSSA infections. The patients with HA-MSSA infections were significantly younger than those with CA-MSSA infections and were significantly associated with the underlying diseases of preterm and neurologic diseases. Significantly more patients with HA-MSSA infections manifested as pneumonia than those with CA-MSSA infections, while more patients with CA-MSSA manifested as SSTI. That nearly 70% of the patients manifesting pneumonia had underlying diseases may be able to explain this finding.

Clinical manifestations of childhood MSSA infection in this study were similar to those for childhood MRSA infection in Taiwan [10]. Whereas, compared to MRSA in Taiwan, which has its dominant clones in each CA- or HA- classification (major clone for CA-MRSA is ST59 while ST239 and ST5 for HA-MRSA) [22, 23], the molecular characteristics of MSSA isolates from pediatric patients in this study were relatively diverse and only one clone had significant difference between CA and HA isolates. Three clones, namely ST188/pulsotype AX, ST15/pulsotype F, ST7 or 8/pulsotype BA, accounted for half of the isolates. These findings were consistent with those in adult patients reported from Taiwan [12, 24, 25], which showed that ST188 was the most common sequence type. The clones of ST7, and ST188 (or *spa* t189) also prevailed in Asian countries such as China [26]. In addition, isolates of ST15/pulsotype F were more frequently seen in CA isolates than in HA isolates with borderline significance (*P* = 0.064) while isolates of ST188/pulsotype AX were significantly more frequently seen in HA isolates (*p* = 0.049). The correlation of the genotype and clinical disease spectrum cannot be identified in this study, possibly due to the relatively small case number of each disease entity.

Panton-Valentine leukocidin (PVL) is a 2-component pore forming toxin (LukS-PV and LukF-PV) targeting phagocytic leukocytes [27, 28] and has been a marked feature of the CA-MRSA. Correlations between PVL and abscess/furuncles are high in epidemiological studies [29] despite animal models [30, 31] do not convincingly link PVL with the pathogenesis of skin lesions. It is noteworthy that PVL genes were identified in 11 MSSA isolates in this study, accounting for 8.4% of the isolates. A previous pediatric study [32] conducted in northern Taiwan showed a PVL harbor rate of 19.5% (14/71) in infecting MSSA isolates from 2003 to 2008. In contrast, 4.17% was reported in China in 2008 [33] and less than 7% in the US in 2004 [34]. In this study, 9 of the 11 PVL-strain were ST59/pulsotype D, whereas the aforementioned Taiwanese pediatric study [32] showed only two ST59/pulsotype D among 19 isolates. Skin and soft tissue infection were the main focus found in PVL-strain, as shown in Table 4. The molecular characterizations of this MSSA strain was similar to those of a dominant local endemic CA-MRSA strain in Taiwan [35]. This endemic CA-MRSA clone, namely MRSA ST59, might be originated from MSSA ST59 with the acquisition of *mecA* gene. A phylogenetic analysis is needed and is ongoing. However, the clinical significance and implication of these PVL-positive MSSA isolates need more studies. In addition, one of the other two PVL-positive isolates was characterized as ST8/t008/pulsotype AI, similar to those of MRSA USA300, which is an endemic CA-MRSA clone prevailing in northern American and emerged in Taiwan recently [36].

Several strains with specific sequencing type deserved more elucidation. Three isolates could not be typed by PFGE and was characterized as ST398, which is a livestock-associated strain and prevailed in Europe [37], not reported from Taiwan previously. The origin of this strain could not be traced in this study. One isolate of pulsotype C were characterized as ST59, also similar to those of local endemic CA-MRSA clones. One isolate of pulsotype-ST30 was shared a similar characteristics of a CA-MRSA clone prevailing in Southeastern Asia [37].

Antibiotics susceptibility test revealed that the susceptibility rate exceeded 90% in most antibiotics except erythromycin, clindamycin and penicillin, which was identical to the finding in MSSA infection in Taiwanese adult population [12]. Comparing to antibiotics susceptibility in Asia and USA, the overall susceptibility rates were higher in Taiwan than in China [38], but the results were similar to pediatric patients in US [39].

There are several limitations in this study. First, this study was conducted in a single medical center and the epidemiologic features shown here may not represent the whole pediatric population in Taiwan. However, our hospital is the largest hospital in Taiwan and the case number in this study was not small, it still can partly reflect the current status of childhood MSSA infection in Taiwan. Second, though the isolates were prospectively collected, medical records of the patients were retrospectively reviewed, some risk factors for MSSA acquisition in the patients may be missed and thus the patients in HA-MSSA group might be misclassified to CA-MSSA group. Third, some of the specimen types e.g. pus, wound swabs, urine and sputum may be easily contaminated by *S. aureus*. Thus, whether or not all *S. aureus* isolates identified in this study caused active infection is somewhat questionable. However, all the isolates were clinical isolates from the patients with active diseases and were treated as such.

## Conclusions

Around one-third of childhood *S. aureus* infections in northern Taiwan were caused by MSSA. SSTIs were the most common manifestation. The molecular characteristics of these clinical MSSA isolates were relatively diverse and with certain significant difference between CA and HA isolates. A small proportion of these MSSA isolates harbored PVL genes.

## Abbreviations

CA: Community-associated
CC: Clonal complex
CLSI: Clinical and Laboratories Standards Institute
CO: Community-onset
HA: Healthcare-associated
HO: Hospital-onset
MLST: Multilocus sequence type
MRSA: Methicillin-resistant *Staphylococcus aureus*
MSSA: Methicillin-sensitive *Staphylococcus aureus*
PFGE: Pulsed-field gel electrophoresis
PVL: Panton-Valentine leucocidin
